# Epidemiologic Features and Environmental Risk Factors of Severe Fever with Thrombocytopenia Syndrome, Xinyang, China

**DOI:** 10.1371/journal.pntd.0002820

**Published:** 2014-05-08

**Authors:** Kun Liu, Ning Cui, Li-Qun Fang, Bing-Jun Wang, Qing-Bin Lu, Wei Peng, Hao Li, Li-Yuan Wang, Song Liang, Hong-Yu Wang, Yao-Yun Zhang, Lu Zhuang, Hong Yang, Gregory C. Gray, Sake J. de Vlas, Wei Liu, Wu-Chun Cao

**Affiliations:** 1 State Key Laboratory of Pathogen and Biosecurity, Beijing Institute of Microbiology and Epidemiology, Beijing, People's Republic of China; 2 The 154 Hospital, People's Liberation Army, Xinyang, People's Republic of China; 3 The Shangcheng People's Hospital, Shangcheng, People's Republic of China; 4 Anhui Medical University, Hefei, People's Republic of China; 5 Environmental and Global Health, College of Public Health and Health Professions, and Emerging Pathogens Institute, University of Florida, Gainesville, Florida, United States of America; 6 Department of Public Health, Erasmus MC, University Medical Center Rotterdam, Rotterdam, The Netherlands; University of Texas Medical Branch, United States of America

## Abstract

**Background:**

Severe fever with thrombocytopenia syndrome (SFTS) is an emerging infectious disease discovered in rural areas of Central China in 2009, caused by a novel bunyavirus, SFTS virus (SFTSV). The disease usually presents as fever, thrombocytopenia, and leukocytopenia, with case-fatality rates ranging from 2.5% to 30%. *Haemaphysalis longicornis* was suspected to be the most likely vector of SFTSV. By the end of 2012, the disease had expanded to 13 provinces of China. SFTS patients have been reported in Japan and South Korea, and a disease similar to SFTS has been reported in the United States.

**Methodology/Principal Findings:**

We characterized the epidemiologic features of 504 confirmed SFTS cases in Xinyang Region, the most severely SFTS-afflicted region in China from 2011 to 2012, and assessed the environmental risk factors. All cases occurred during March to November, with the epidemic peaking from May to July. The patients' ages ranged from 7 to 87 years (median 61 years), and the annual incidence increased with age (χ^2^ test for trend, P<0.001). The female-to-male ratio of cases was 1.58, and 97.0% of the cases were farmers who resided in the southern and western parts of the region. The Poisson regression analysis revealed that the spatial variations of SFTS incidence were significantly associated with the shrub, forest, and rain-fed cropland areas.

**Conclusions:**

The distribution of SFTS showed highly significant temporal and spatial heterogeneity in Xinyang Region, with the majority of SFTS cases being elderly farmers who resided in the southern and western parts of the region, mostly acquiring infection between May and July when *H. longicornis* is highly active. The shrub, rain-fed, and rain-fed cropland areas were associated with high risk for this disease.

## Author Summary

Severe fever with thrombocytopenia syndrome (SFTS) is an emerging infectious disease discovered in rural areas of Central China in 2009, caused by a novel bunyavirus, SFTS virus (SFTSV). The disease usually presents as fever, thrombocytopenia, and leukocytopenia, with case-fatality rates ranging from 2.5% to 30%. By the end of 2012, the disease had expanded to 13 provinces of China. SFTS patients have been reported in Japan and South Korea, and a disease similar to SFTS has been reported in the United States. Here we characterized the epidemiologic features of 504 confirmed SFTS cases in Xinyang, the most severely SFTS-affected region in China from 2011 to 2012, and identified the environmental risk factors. We found the distribution of SFTS cases showed highly significant temporal and spatial heterogeneity, with the majority of SFTS cases being elderly farmers who resided in the southern and western parts of the region, mostly acquiring infection between May and July when *H. longicornis* is highly active. The shrub, forest, and rain-fed cropland areas were strongly associated with high risk for SFTS.

## Introduction

Severe fever with thrombocytopenia syndrome (SFTS) is an emerging infectious disease discovered in middle-eastern China [1]. The disease usually presents as fever, thrombocytopenia, and leukocytopenia, with case-fatality rates ranging from 2.5% to 30%. In 2009, the causative agent was identified as a novel bunyavirus in the genus of *phlebovirus*, family *Bunyaviridae*, and designated as the SFTS virus (SFTSV) [1]–[4]. Immediately after noticing the epidemic, the Chinese Ministry of Health initiated a national surveillance program [5]. By the end of 2012, SFTS cases had been reported in 13 provinces of China [6]. Most recently, SFTS patients have been reported in Japan and South Korea and a disease similar to SFTS has been reported in the United States [7]–[9].The potential for SFTS to spread to other countries of the world, in combination with its high fatality rate, possible human-to-human transmission, and extensive prevalence among residents and domesticated animals in endemic regions [10]–[16] make the disease a severe threat to public health.

SFTSV has been detected and isolated from *Haemaphysalis longicornis* ticks in the endemic areas. The high sequences homology between viruses isolated from ticks and those from patients suggested this tick species as the most likely vector [1]–[4]. It's long been recognized that many tick-borne diseases such as Lyme disease, tick-borne encephalitis, and rickettsiosis are zoonotic and have shown strong associations with environmental elements [17]–[21]. We hypothesize that the environmental factors might also contribute to the occurrence and distribution of SFTS. However, no report has explored the environmental factors associated with this emerging infectious disease. The role of environmental factors in human infection with SFTSV remains unclear. The objectives of this study were to characterize the epidemiologic features and to identify the environmental risk factors of the disease in one of the most severely affected regions by the disease.

## Materials and Methods

### Study Site

The study was performed in Xinyang, an administrative region of Henan Province in middle-eastern China located between113°42′–115°56′E and 31°23′–32°40′N (online Technical Appendix Figure S1). Xinyang reported 99% of SFTS cases in Henan Province [22] and 48% of SFTS cases in China [23]. The region includes 200 administrative townships of 10 counties and districts, with a total area of 18,819 square kilometers and a population of 6,108,683 residents. Xinyang has a humid subtropical climate with annual precipitation of around 1,100 millimeters. The region is characterized by its distinct natural landscapes, with the northern part mainly comprising plains and the southern part stretching across the Dabie Mountain range.

### Data Collection and Management

From January 1, 2011 to December 31, 2012, laboratory-confirmed SFTS cases in Xinyang Region were included in the analysis. According to the national guidelines [5], a laboratory-confirmed SFTS case was defined as meeting one or more of the following criteria: 1) a positive SFTSV culture; 2) a positive result for SFTSV RNA by molecular detection; 3) seroconversion or ≥4-fold increase in specific antibody to SFTSV between acute and convalescent serum samples. Information regarding age, sex, occupation, onset date of symptoms, and residential address were collected. Each case was geo-referenced to a digital map of Xinyang Region according to his or her residential addresses assuming they had never left their living place in the last two weeks before onset of symptoms.

To explore the relationship between the SFTS incidence and the environmental factors, the data regarding land cover, normalized difference vegetation index (NDVI) and elevation were collected and processed. Land cover data were derived from a raster version of “GlobCover 2009 land cover map” (available at http://ionia1.esrin.esa.int), which was processed by the European Space Agency [24]. Land cover types were classified as follows: irrigated cropland, rainfed cropland, orchard, forest, shrub, built-up land and water body. For each type of land cover, its covering proportion of each township was calculated using ArcGIS 9.3 software (ESRI Inc., Redlands, CA, USA). NDVI, which represents the amount and productivity of vegetation [25], was derived from “Free Vegetation Products” (http://free.vgt.vito.be), then the average value in each township was calculated in ArcGIS 9.3. Elevation data were obtained from Shuttle Radar Topography Mission (SRTM) archives (http://www.srtm.csi.cigar.org). Demographic data were obtained from the Xinyang Bureau of Statistics from the sixth national census in 2010, and the average population density for each township was calculated.

### Ethics Statement

The research protocol was approved by the human ethics committee of hospitals where the study was performed (including the 154 Hospital of People's Liberation Army, the Shangcheng People's Hospital, the Xinxian People's Hospital) and the institutional review board of State Key Laboratory of Pathogen and Biosecurity, Beijing Institute of Microbiology and Epidemiology. All participants provided written informed consent, for the cases of children, parents or guardians of eligible children were informed and asked to provide written informed consent on behalf of their children. The study-related information was analyzed anonymously.

### Statistical Analysis

We applied Poisson regression to explore the association between SFTS incidence and environmental factors at the township level, using STATA 10.0 software (StataCorp LP, College Station TX, USA). The variables considered in the analysis included land cover, elevation, NDVI and population density.

Univariate Poisson analysis was employed for each variable. The variables with a *P*-value <0.10 in the univariate analysis were included in the multivariate analysis. For all continuous variables, we also presented trisection categorical results to inspect whether or not the assumption regarding continuous variables was justified [26]. A scale parameter was applied to compensate for the over-dispersion, and the collinearity between covariates was assessed. The percentage change (PC) in incidence in response to the change of a variable by a given amount, 95% confidence intervals (CIs), *P*-values were estimated after correction for over-dispersion, and a *P*-value <0.05 was considered to be significant.

## Results

**Figure 1:**
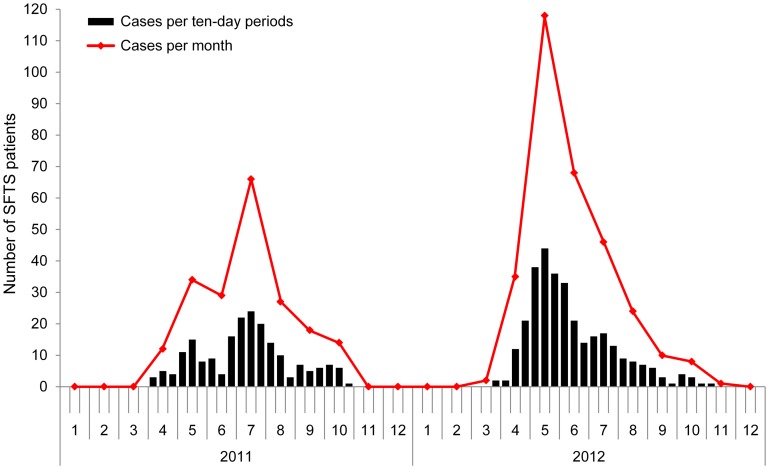
Temporal distribution of the confirmed SFTS cases in Xinyang Region, 2011–2012. The black histogram represents the number of SFTS cases per period of ten days, and the red line represents the monthly number of SFTS cases.

A total of 504 laboratory-confirmed SFTS cases (193 in 2011 and 311 in 2012) were reported. All of them occurred during March to November (Figure 1), with epidemic peaking from May to July (71.6%, 361/504). In 2011, case number peaked in July (32.1%, 62/193), while in 2012, the peak occurred in May (37.9%, 118/311).

**Figure 2:**
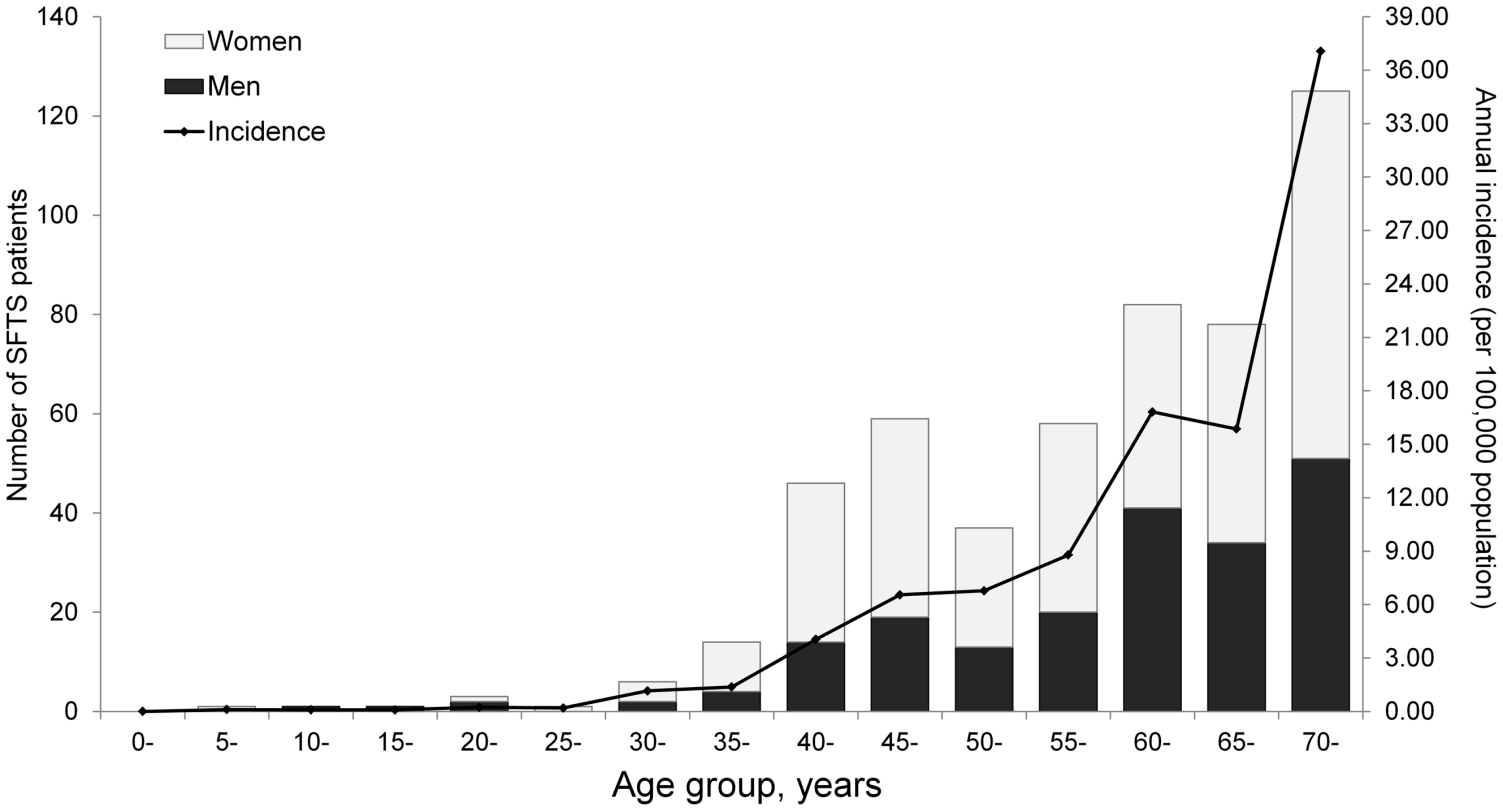
Age and gender distribution of the confirmed SFTS cases in Xinyang Region, 2011–2012. The black histogram represents the number of male cases and the white histogram represents the number of female cases over 5-year age groups. The line represents the annual average incidence (1/100,000) over age groups.

The patients' ages ranged from 7 to 87 years (median 61 years) old, and the mean (±SD) age was 59.4 (±12.9) years. Age distribution demonstrated that the annual incidence increased with age (χ^2^ test for trend, P<0.001) (Figure 2).The female-to-male ratio of cases was 1.58. Overwhelming majority of confirmed cases lived in rural areas, and 97.0% (489/504) of the cases were farmers being engaged in agriculture activities. All the recruited cases in the current study did not report infection through human-to-human transmission.

**Figure 3:**
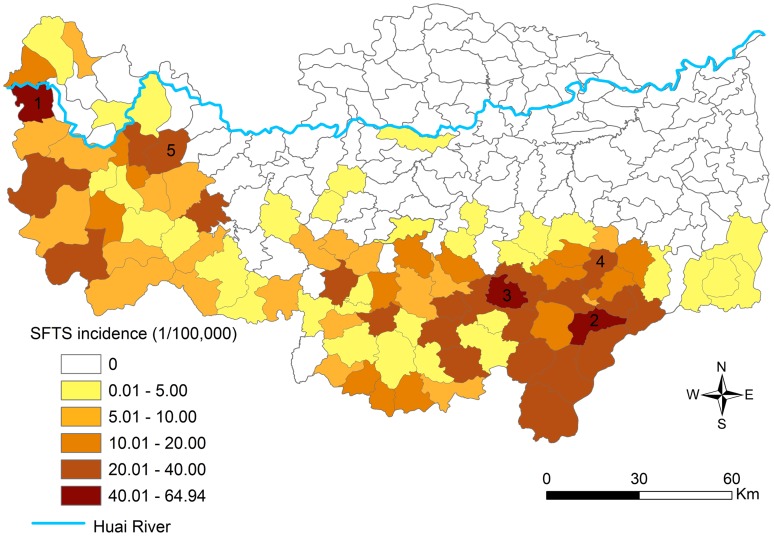
The annual incidence of confirmed SFTS for each township in Xinyang Region, 2011–2012. The top five incidences displayed in the figure were 64.94, 56.93, 43.32, 38.84, and 38.00 per 100,000 persons in the townships of Gaoliangdian, Wanggang, Guanmiao, Hefengqiao, and Yanghe, respectively.

The annual incidence tremendously varied from township to township ranging from 0 to 64.9 per 100,000 people, with an average of 4.2/100,000 people in the study site. The geographic distribution of annual SFTS incidence is displayed in the thematic map (Figure 3), twenty-nine of 200 townships in the southern and western parts of Xinyang Region had the annual incidences over 20.0/100,000. The 5 townships with highest incidences were Gaoliangdian, Wanggang, Guanmiao, Hefengqiao and Yanghe. No case was found in 102 townships in northern Xinyang.

**Figure 4:**
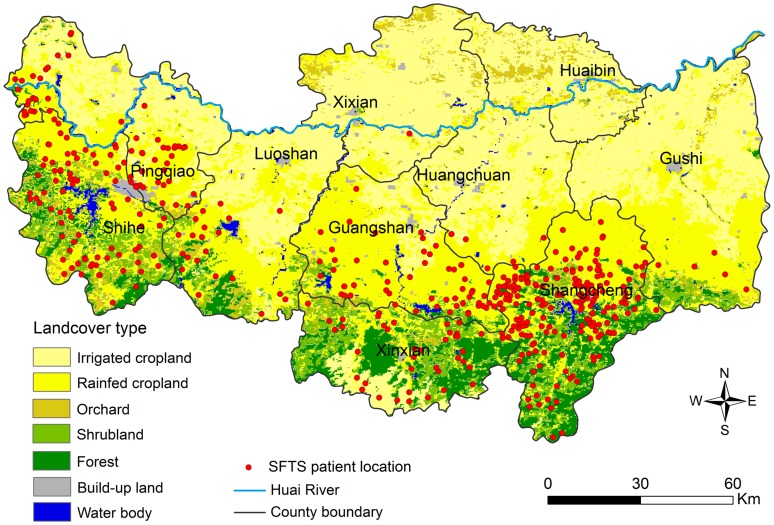
Spatial distribution of the confirmed SFTS cases overlapping the map of land cover in Xinyang Region, 2011–2012. Seven land cover types have been categorized in the study area: irrigated cropland, rainfed cropland, orchard, forest, shrub, built-up land, and water body. Irrigated cropland comprises fields under irrigation for planting crops. Rainfed cropland represents areas with rainfed herbaceous crops. Orchard includes cultivated and managed terrestrial areas. Forest areas comprise broad-leaved forest and coniferous forest. Shrub areas included shrubland, grassland, and isolated trees. Built-up land areas concern surfaces with buildings and associated areas. Water body areas comprise lakes, reservoirs and ponds.

Based on the univariate analysis, six variables (shrub, forest, irrigated cropland, rainfed cropland, orchard, and elevation) were significantly associated with SFTS incidence (Table 1). The multivariate analysis revealed that SFTS incidence was raised with increases in proportion of shrub and forest (Table 1). The association between SFTS incidence and proportion of rainfed cropland showed an inverted-U pattern relationship. With the rainfed cropland proportion increasing, SFTS incidence rose to the peak and then dropped (Table 1). Elevation was removed from the multivariate analysis because of its collinearity with forest (r = 0.89).The results coincided with the spatial distribution shown in Figures 3 and 4, where SFTS cases predominantly occurred in the southern and western forest, shrub, and the surrounding rainfed cropland areas, while cases were rarely reported in the northern and eastern plains.

**Table 1:** Poisson regression analysis of environmental factors associated with SFTS incidence in Xinyang Region, China, 2011–2012.*

Variables (Unit)†	Annual average incidence (95% CI)	Univariate analysis		Multivariate analysis	
		Crude PC (95% CI)	P-Value	Adjusted PC (95% CI)	P-Value
Shrub (categorical, 1%)					
<0.1	0.28 (0.17, 0.44)				
0.1–9.9	2.26 (2.15, 2.38)				
≥10.0–100	15.05 (13.74, 16.35)				
Shrub (continuous, 10%)		68.76 (48.76, 91.44)	<0.001#	51.22 (31.56, 73.82)	<0.001
Forest (categorical, 1%)					
<0.1	0.55 (0.47, 0.63)				
0.1–9.9	8.07 (7.79, 8.34)				
≥10.0–100	18.20 (16.34, 20.07)				
Forest (continuous, 10%)		55.13 (41.66, 69.89)	<0.001#	51.33 (32.98, 72.21)	<0.001
Irrigated cropland (categorical, 1%)					
<3.0	12.29 (11.13, 13.44)				
3.0–69.9	3.46 (3.34, 3.59)				
≥70.0–100	0.45 (0.41, 0.51)				
Irrigated cropland (continuous, 10%)		−29.87 (−36.68, −22.33)	<0.001#	NS (excluded)	
Rainfed cropland (categorical, 1%)					
<16.0	3.52 (3.07, 3.98)				
16.0–59.9	7.09 (6.31, 7.73)				
≥60.0–100	4.46 (4.20, 4.77)				
Rainfed cropland (continuous, 10%)		77.07 (29.18, 142.70)	<0.001#	90.42 (37.49,163.72)	0.001
Quadratic rainfed cropland (continuous, 1%)		−5.73 (−8.73, −2.64)	<0.001#	−4.62 (−7.77, −1.36)	0.006
Orchard (categorical, 1%)					
<2.0	4.15 (3.63, 4.66)				
2.0–6.9	5.11 (4.25, 5.71)				
≥7.0–100	7.37 (6.66, 8.06)				
Orchard (continuous, 10%)		37.48 (2.29, 93.45)	0.022#	NS (excluded)	
Built-up land (categorical, 1%)					
<0.1	7.19 (6.57, 7.77)				
0.1–2.9	2.46 (2.45, 2.47)				
≥3.0–100	2.08 (1.88, 2.28)				
Built-up land (continuous, 10%)		−52.35 (−84.68, 48.16)	0.200		
Elevation (categorical, 10 m)					
<5.30	0.17 (0.17, 0.17)				
5.30–9.59	3.02 (2.87, 3.18)				
≥9.60	13.40 (12.24, 14.56)				
Elevation (continuous, 10 m)		6.45 (4.83, 8.09)	<0.001§		
NDVI (categorical, 0.1)¶					
<4.7	4.20 (4.06, 4.48)				
4.7–5.4	6.81 (6.16, 7.46)				
≥5.4–10.0	5.38 (4.35, 6.39)				
NDVI (continuous, 0.1)		13.06 (−23.16, 66.34)	0.533		
Population density (continuous,100/km^2^)		−3.57 (−69.26, 202.47)	0.958		

*SFTS = severe fever with thrombocytopenia syndrome;

¶ NDVI = normalized difference vegetation index;

NS, not significant.

† For all continuous variables, we also reported categorical results to permit inspection of the data and whether or not the assumption of continuous variables was justified. For categorical variables, the number represented proportion of land cover areas in the township.

# Variable that was included into multivariable analysis.

§ Elevation was removed from the multivariate analysis because of its collinearity with forest (r = 0.89).

## Discussion

In the current study, we provide an overview of the epidemiologic features of the novel human bunyavirus infection in Xinyang, the most severely SFTS-affected region in China. Highly significant temporal and spatial heterogeneity of the disease was identified, with the majority of SFTS cases being elderly farmers who resided in the southern and western parts of the region, mostly acquiring infection between May and July. The shrub, forest, and rainfed cropland areas were significantly associated with high risk for SFTS.

Since the disease was discovered in 2009, ticks have been considered to be the most likely vector. People who live in mountainous or hilly rural areas were suggested to be the high-risk populations [1], [2], [4]. Our epidemiologic results corroborated the current knowledge on the epidemiology of SFTS. According to our results, 97.0% of confirmed patients were farmers being engaged in agriculture activities, with some reporting tick bites within 2 weeks before the symptom onset. We also observed a high incidence of SFTS among people over the age of 60 years old, and more females than males among the cases. We hypothesized the age and gender specific distribution of the disease might be related with exposure characteristics of the local population. In Xinyang Region, most young people take industrial work, instead of farming activity. In contrast, the elderly take the main agriculture activities (preparing land for cultivation, planting crops, pasturing cattle, and clearing weeds, etc), especially tea-picking activity, which was performed mostly by elderly women from May to July when *H. longicornis* is highly active in this region [2], [22], [27]. This high exposure experience in elderly, especially in female could remarkably increase the risk for SFTSV infection. On the other side, we could not determine whether the waning immunity of the elderly might also play a role in the age specific distribution of the disease, since the population immunity level was not evaluated. Based on this hypothesis, people living in the endemic regions should be aware of the main causes of exposure, and self-protective measures should be taken to avoid being bitten by ticks accordingly.

Using the Poisson regression analysis, we found that shrub, forest, and rainfed cropland showed strong associations with SFTSV infection. These findings may help us to explain spatially-clustered distribution of SFTS cases. The risk of SFTS incidence rose linearly with increasing shrub and forest areas. However, the relationship between SFTS incidence and rainfed cropland area showed an inverted-U pattern relationship (Table 1). This finding was consistent with the previous survey in Xinyang [27]. Liu et al. described shrub and forest areas as ideal habitats for *H. longicornis*. It seems quite possible that geographic expansion of this tick population plays a role in SFTS spreading in the region. Despite of these associations, the exact role of ticks and possible wild animal reservoirs for SFTSV merit future well-designed tick transmission competence studies.

We also recognize limitations of the study. First, the hospital-based surveillance captured data only from patients with SFTSV who sought medical care. As patients with subclinical infection might have been missed, our data do not offer complete SFTSV disease spectrum and epidemiological characteristics. Second, we only considered major potential environmental factors into our statistical analysis. Climatic factors were not studied because meteorological data were unavailable. Furthermore, data for other potential factors such as population immunity, economic conditions, ticks density, etc were not included in the study, which need further investigation.

In conclusion, we characterized the epidemiologic features of SFTS cases in Xinyang Region, and demonstrated that shrub, forest, and rainfed cropland areas were associated with high risk of SFTS incidence. As no vaccine against SFTS is available, and fatal outcomes are common, our findings can be used to identify high risk areas and populations, which might assist public health officials in developing and targeting educational programs and other interventions to reduce the disease incidence.

## Supporting Information

Figure S1 **The location of study site in China.**
(TIF)

Checklist S1 **STROBE Checklist.**
(DOC)
