# Supplementary figures and images for: Epidemiologic Features and Environmental Risk Factors of Severe Fever with Thrombocytopenia Syndrome, Xinyang, China
Source: PLoS Negl Trop Dis. 2014 May 8;8(5):e2820. doi: 10.1371/journal.pntd.0002820 (PMC4014392; doi:10.1371/journal.pntd.0002820)

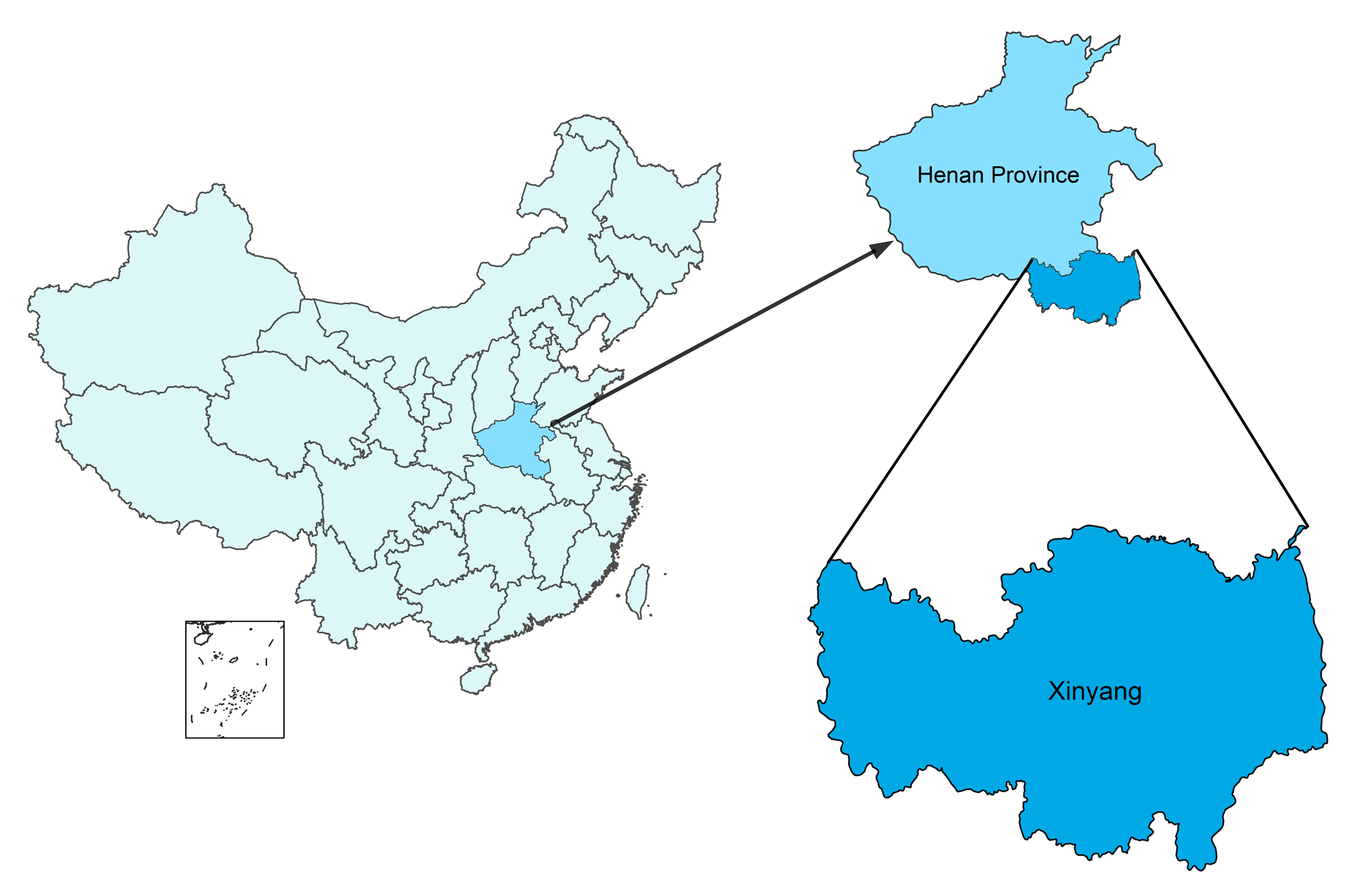

Supplement: Figure S1 — The location of study site in China. (TIF) [file pntd.0002820.s001.tif]
